# Holocene landscape evolution and its interaction with human activities in the southern piedmont of Taihang Mountain, Central China

**DOI:** 10.3389/fpls.2022.980840

**Published:** 2022-09-23

**Authors:** Xiaohu Zhang, Shugang Yang, Peng Lu, Yaping Li, Panpan Chen, Zhengkai Xia

**Affiliations:** ^1^Henan Provincial Institute of Cultural Relics and Archaeology, Zhengzhou, China; ^2^Henan Provincial International Joint Laboratory on Origins of Modern Humans in East Asia, Zhengzhou, China; ^3^Institute of Geography, Henan Academy of Sciences, Zhengzhou, China; ^4^Institute of Cultural Heritage, Shandong University, Qingdao, China; ^5^Laboratory for Earth Surface Processes, Ministry of Education, College of Urban and Environmental Sciences, Peking University, Beijing, China

**Keywords:** Taihang Mountain, the Holocene, pollen record, landscape evolution, human activities

## Abstract

Piedmont zones have been witnessing intensive human activities since ancient times. However, it remains unclear when it comes to the environmental mechanism for early humans exploiting piedmont zones. Here we present a case study about the interactions between early human activities and landscape evolution in the piedmont of Taihang Mountain, an area with prominent ecological and cultural significance in Chinese history. Based on chronological and pollen analyses, we reconstruct the regional landscape evolution in the Fengtougang (FTG) site of southern Taihang Mountain during the Holocene. The results show that the area has been dominated by terrestrial plants since the late Longshan culture (4000 BP), including a large number of *Pinus, Artemisia, Spiraea*, and *Gramineae*, a few *Cattails*, and some other aquatic herbs. During the early history (4000-2000 BP), there is a combination of *Pinus, Artemisia, Spiraea, Compositae*, and *Selaginella Chinensis*, with a few aquatic plants. Since the late history (500 BP), the *Chinese selaginella, Pinus, Selaginella*, and *Sedge families* dominate, with no aquatic plant pollen found. Combining the detailed geoarchaeological survey, grain size analysis, and magnetic susceptibility analysis, we demonstrate that there should be a landscape of extensive floodplain during the early-middle Holocene (10000-4000 BP). During the late Longshan culture (about 4000 BP), the study area should be dominated by a landscape of sparse forest grassland with interlacing rivers and lakes. With river downcutting and watercourse fixation since the late Longshan culture, the flooded area massively shrinks, providing suitable habitat for human settlement. From then on, human activities begin to move to the study area on a large scale, followed by continuous cultural development and thriving early civilization.

## Introduction

The piedmont zone usually refers to the transition area from mountain to plain. These areas are generally composed of alluvial fans, tablelands, and inclined plains. The terrain is relatively flat and wide, and the relative elevation difference is low. The piedmont zone has always been an area with intensive human activities. For many famous mountains in the world, such as the Andes in South America (Garcia et al., 1999; Shimada et al., 1991; Wilson et al., 2022), the Alps in Europe (Tinner et al., 2000), and the Mount Song in China (Zhou et al., 2005; Lu et al., 2012; Wang et al., 2015; Yan et al., 2019; Tang et al., 2019), their piedmont zones are all associated with concentrated human settlements and cities in both ancient and present times. This is mainly attributed to the favorable natural environment in such areas, including the wide flat terrain, abundant water resources, and diversified biological resources.

Large-scale human activities are not inherently consistent in the piedmont zone throughout history. Some scholars claim that during the Paleolithic, Neolithic, and Bronze Ages, the main residence place of humans continuously changes from the mountainous region to the tableland and subsequently the piedmont (Zhou et al., 2005). Nonetheless, it remains ambiguous about the timing that humans start to massively exploit the piedmont zone and the factors that trigger the large-scale human activities in the piedmont zone. In fact, the residence place for humans is determined jointly by multiple factors, such as the natural environment, subsistence strategies, technical levels, and cultural characteristics (Lu et al, 2017a; Lu et al., 2019). In the prehistoric period associated with low productivity, the location of human settlement is considered to be more closely related to the natural environment (Lu 2017b; Lu et al., 2021). Accordingly, reconstructing the early landscape provides an important approach to probing into the mechanism of the large-scale human activities in the piedmont zone.

Since 2014, we have carried out systematic archaeological surveys in the southern piedmont of Taihang Mountain. We also carefully analyze the temporal and spatial distributions of the early settlement, vegetation, hydrology, landform, and sedimentary structure. The Fengtougang (FTG) site, a representative human settlement, is selected for archaeological excavation to further confirm the cultural characteristics. Samples are collected from geological sections with continuous deposition and abundant environmental information to perform the chronological analysis and paleo-environmental reconstruction. Our goal is to understand the characteristics and processes of human activities and landscape evolution in the piedmont of Taihang Mountain through these analyses.

## Regional setting

**Figure 1:**
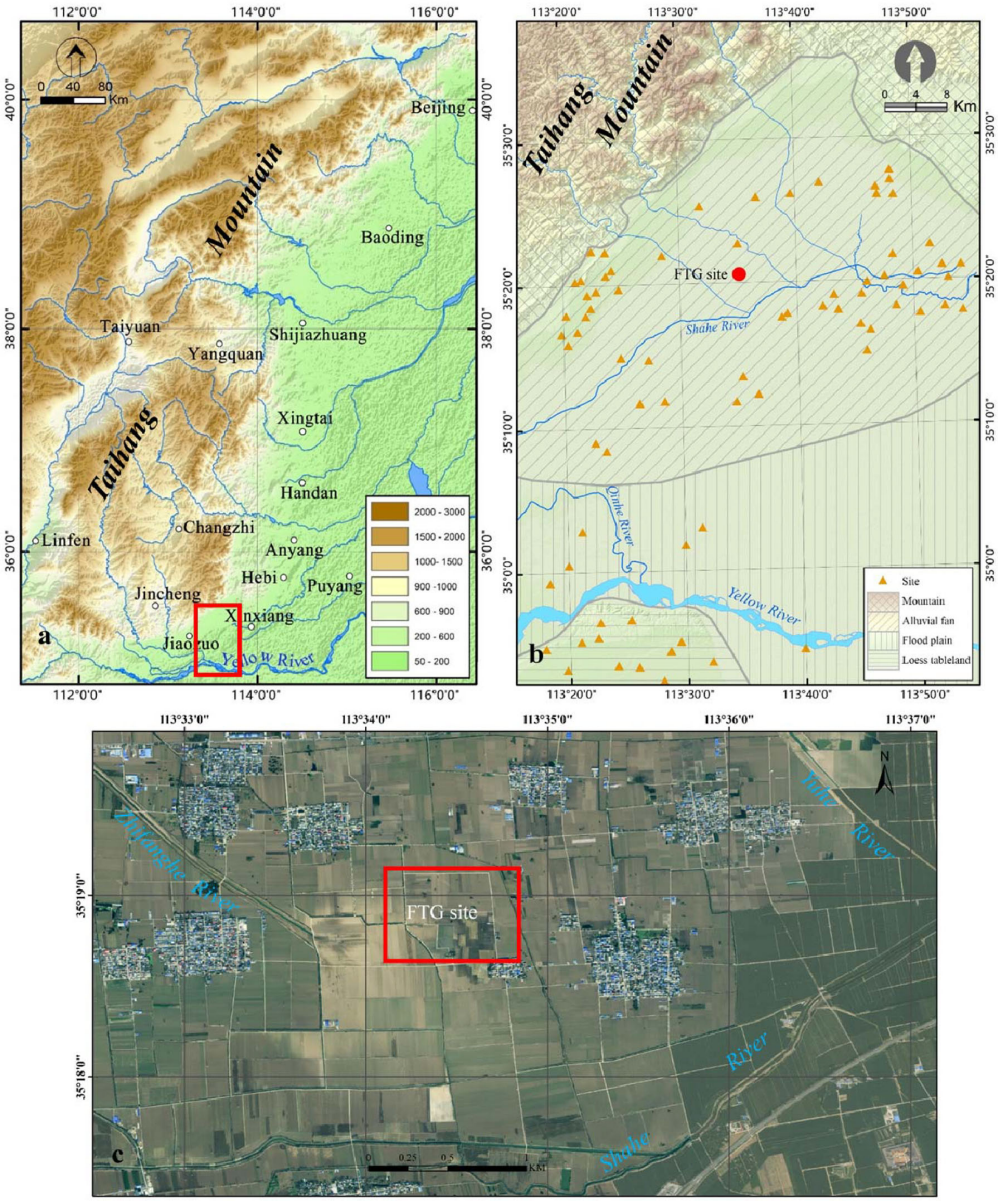
The study area **(A)**. There are a lot of ancient and modern cities in the piedmont of Taihang Mountain, which is our study area and has been marked with a red rectangle; **(B)** The geomorphic types and Neolithic-Bronze sites in the study area. The red circle represents the location of the FTG site; **(C)** The terrain and river system in the FTG site area. The image comes from Map World.).

As an important geographic boundary between the Loess Plateau and the North China Plain (You and Yang, 2013), Taihang Mountain divides the semi-arid and semi-humid areas of China (Zheng, 2017). Moreover, its piedmont zone has been an area of intensive human activities since ancient times, witnessing the development of many famous cities, including Beijing, the Anyang Yin Ruins, and the Taosi site (**Figure 1A**).

Our study area is located in the southern piedmont of Taihang Mountain, geographically involving Huixian, Huojia, Xiuwu, and Wuzhi Counties in Henan Province (**Figure 1B**). The regional terrain is high in the north and low in the south, with an altitude of 1000-50 m. The region is located within the warm temperate continental monsoon climate zone, with an annual average temperature of 14°C and an average annual precipitation of 600 mm. The regional soil is dominated by brown earth and fluvo-aquic soil. Vegetation is the warm-temperature broadleaf deciduous forest. However, crops and artificial vegetation are prevalent in many areas, due to the long-lasting agricultural activities. There are numerous rivers in the region. Besides a few rivers belonging to the Yellow River system, most of them flow eastward into the Hai River finally.

The southern Taihang region is also a key region when it comes to the origin of the Chinese civilization. The regional Neolithic-Bronze cultures can be divided into the Peiligang period (9000-7000 BP), Yangshao period (7000-5000 BP), Longshan period (5000-4000 BP), Xia-Shang period (4000-3000 BP), and Zhou Dynasty (3000-2200 BP). Although the southern Taihang region is associated with representative early cultures and environmental characteristics, research on the Holocene landscape is rare. The studies on the interaction between human activities and environmental evolution are even fewer (Cao, 1994; Wang, 2009; Xu et al., 2013; Zhai et al., 2019).

According to the landform, altitude, material composition, and sedimentary structure, four geomorphic types are identified in the study area, namely mountains, loess tablelands, alluvial fans, and floodplains. Mountains and loess tablelands are of limited distribution, while alluvial fans and floodplains are prevalent (**Figure 1B**). The floodplain is mainly located in the southern area and is formed by the recurrent alluviation of the Yellow River, Qinhe River, and their tributaries. The top of the plain is flat and wide, with an altitude of about 80 m. The sediments are mostly alluvial deposits of various ages. The alluvial fan is mostly located in the northern-central region. The landform slopes from north to south, with altitudes of 150–80 m. Numerous Neolithic-Bronze Age settlements are discovered in the piedmont zone during the archaeological investigation, but only a few of them have been systematically excavated and studied (Yuan, 2000; Zhao, 2010). Atlas of Chinese Cultural Relics (NCHAC, 1991) shows that except for several sites containing remains of the Yangshao culture, most sites are attributed to the Longshan culture. Nonetheless, the region lacks chronological data of relevant sediments and cultural relics. Most ages are determined according to pottery shards scattered across the surface.

The FTG site is one of the early settlements distributed in the southern Taihang piedmont (**Figure 1C**). Its geographical coordinates are E113°34′20″, N35°18′50″, with an altitude of about 80 m. The site area is 32,000 m^2^ and the cultural deposits are 4 m thick. The south of the FTG site is adjacent to the Dasha River, a tributary of the Hai River, while the Yu River originating from Taihang Mountain flows in the east of the site. Furthermore, the FTG site is identified to lie at the margin of the alluvial fan of the Taihang piedmont. The archaeological investigation indicates that the FTG site is dominated by the Longshan culture, with a few remains of the Shang Dynasty, Warring States, and Han Dynasty (NCHAC, 1991).

## Material and methods

### Strata at the FTG site

**Figure 2:**
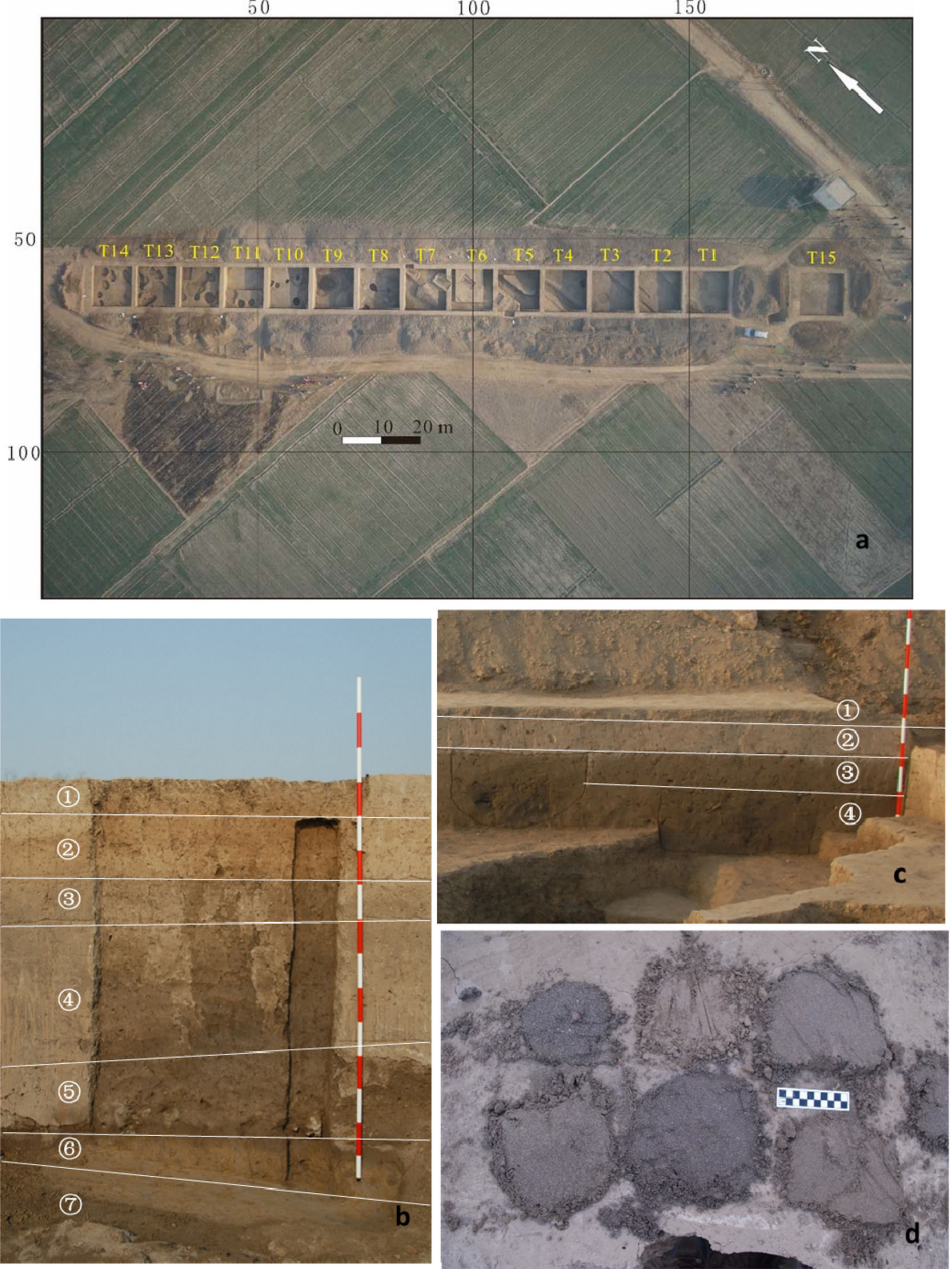
Structure and spatial distribution of the Late Quaternary deposits in the FTG site. **(A)**. The distribution of excavation grids in the FTG site. The lacustrine facies strata are mainly distributed in T1-T9, while T10-T14 are primarily the cultural deposits of ancient human activities; **(B)** The east wall section of T7; **(C)** The east wall section of T13; **(D)** The archaeological drilling exploration shows that both basements of T7 and T13 are a set of grey-yellow silt-fine sands, which form the bottom deposits in the site.).

Since the Neolithic-Bronze settlements in the region are mostly discovered in the middle and periphery of the alluvial fan, landscape evolution in these areas is essential to understanding the adaption process of early humans in the piedmont zone. In 2014, we started to excavate the FTG site located on the alluvial fan edge. Fifteen archaeological grids (10 m by 10 m) in total (numbered T1–T15 respectively) are excavated, with an excavation area of 1500 m^2^ (**Figure 2A**).

The excavation shows that two distinctive sets of deposits are present in the FTG excavation region. One is the human cultural deposit in the northern excavation sites, represented by T10–T14; the other is the grey-brown or grey-black silty deposit in the southern excavation sites, represented by T1–T9. Here the west wall sections of the T7 and T13 are taken as examples to introduce the deposits of different areas.

#### The west wall section of T7

The west wall section of T7 is excavated to 240 cm below the surface and the deposits can be divided into seven layers (**Figure 2B**).

**Layer ①:** 0.18–0.3 m thick, light grey-brown silts, unconsolidated with numerous inclusions of plant root systems and a few charcoal debris and pottery shards. This layer represents the modern cultivated deposits.

**Layer ②**: 0.3–0.5 m thick, dominant light brown silts, with some red-brown silts. This layer is seen with a small amount of charcoal debris and consolidated burnt soils. A few blue-and-white porcelain chips and fragments of plate tiles and black bricks are included. According to the contained remains, this layer represents the deposits of the Ming-Qing Dynasty.

**Layer ③**: 0–0.04 m thick, grey clays. It is relatively consolidated, with some yellow-brown vertical wormholes, numerous small calcareous nodules, burnt soils, and a few small gravels. The thickness of this layer is uneven, and the layer gradually thins from south to north and eventually pinches out. Some local positions are seen with many plate tiles, semicircle-shaped tiles, and pottery shards. This layer represents the deposits formed during the Eastern Zhou Dynasty.

**Layer ④**: 0.35–0.85 m thick, grey-brown clays, with some aquatic shells and well-developed calcareous nodules (mostly 4–5 mm in size, with the largest up to 4–5 cm in size). The color of these deposits becomes lighter vertically upward. A few pottery shards, animal bones, stone artifacts, and bone artifacts of the Shang Dynasty are discovered.

**Layer ⑤:** about 0.3 m thick, dark grey-brown clays that appear to be black. Notable calcareous nodules, mostly 1 cm in size and occasionally up to 4–5 cm, are observed, with some burnt soils and a few pottery shards of the Longshan culture.

**Layer ⑥:** about 0.2 m thick, grey-yellow silty-fine sands with calcareous nodules.

**Layer ⑦**: about 0.9 m thick, dark-brown medium-coarse sands that appear to be black. This layer presents a lentoid distribution and contains a few small gravels and well-developed calcareous nodules up to 17 cm in size.

#### The west wall section of T13

The deposits in this section are about 2 m thick and consist of 4 layers (**Figure 2C**).

**Layer ①:** about 0.2 m thick, grey-brown silty clays with numerous plant root systems. It represents the modern agricultural deposits.

**Layer ②:** about 0.24–0.4 m thick, light brown silts that appear to be red-brown. It is relatively consolidated and contains a few charcoal debris and burnt soils. A small number of Ming-Qing Dynasty porcelain chips, grey pottery shards, and black bricks are observed. It represents the deposits formed during the Ming-Qing Dynasty.

**Layer ③**: about 0.3 m thick, unconsolidated light grey silts, with a few charcoal grains and burnt soils. Many pottery shards are present, with identifiable artifacts. This layer represents the cultural deposits of the late Shang Dynasty and the remains of the Shang Dynasty and Longshan culture (e.g. ash pits) are found below the layer.

**Layer ④**: about 1 m thick (evacuated), silty-fine sands with colors varying from grey-yellow to yellow-brown. It contains a few calcareous nodules and some black brown vertical wormholes are developed in the upper part. It represents the deposits before human activities.

The deposit characteristics indicate that the Layers ③, ④, and ⑤ of T7 are deposits of the limnetic facies, distributed from the southernmost T1 to the intersection between T11 and T12 in the north. Archaeological drilling shows that the base of the limnetic deposits is a set of grey-yellow silt-fine sands, which complies with Layer ④ of the T13 west wall, and below these sands are dark brown medium-coarse sands (**Figure 2D**). The distribution of these deposits extends northward and is overlain by the cultural remains in the northern excavation sites.

### Dating

Since the investigated geological sections are within the archaeological site and contain many cultural remains, archaeologists can determine the age of each layer. To confirm the precision of the archaeological dating, chronological samples are collected from Layers ③, ④, and ⑤ of the west wall of the T7 to perform the radioactive ^14^C dating. The chronological dating is performed at the BETA Laboratory using the accelerator mass spectrometer (AMS). The half-life period of the ^14^C is 5568 years. The BP in the dating results means the number of years before the year of 1950. The calibration curve is IntCal20 using the OxCal 3.10 program.

### Pollen analysis

To reconstruct the regional vegetation landscape (Xu et al., 2007; Chen et al., 2015; Ren et al., 2019; Wang et al., 2019; Zhang et al., 2020), 23 sedimentary samples are collected from the T7 west wall section at an interval of 4 cm to perform the pollen analysis. The tests are completed at the Research Center of Paleontology and Stratigraphy (RCPS), Jilin University, China. Fifty grams (dry weight) of each sample is taken and mixed with about 9550 lycopodium spores for calculating the sporopollenin concentration. Then, the mixture is treated successively with hydrochloric acid, hydrofluoric acid, and hydrochloric acid, after which the pollen of samples is concentrated in the test tube *via* a screening process. Two sections are prepared for identification and counting using the biological microscope. Since the quantity of pollen in the deposits is low, it is required to count more than 100 grains of pollen in total for each sample. It is difficult to accurately reveal the history of regional climate change due to the small amount of palynology. Therefore, the pollen results are only used as a reference for regional vegetation landscape reconstruction. Besides the total pollen quantity, the lycopodium spore is also counted and the total pollen concentration of each sample is computed using the concentration conversion formula.

### Analysis of grain size and magnetic susceptibility

At an interval of 2 cm, 56 sedimentary samples are collected from the T7 west wall from bottom to top for analyzing the grain size and magnetic susceptibility.

The grain size measurement is accomplished at the Digital Environmental Archaeological Laboratory, Institute of Geographical Sciences, Henan Academy of Sciences. The samples are treated with hydrogen peroxide and diluted hydrochloric acid to remove organic matter and carbonates, respectively. Then, 10 mL of 0.05 mol/L sodium hexametaphosphate is added. The sample is heated until boiling and tested after the grains are sufficiently dispersed. The test instrument is Malvern Mastersizer 2000E laser-based grain size analyzer. For each sample, the test is repeated either three times or until the reproducible grain size distribution curve is observed, if necessary. The following-presented results are the averages of multiple tests of each sample.

The magnetic susceptibility test is finished at the Environmental Archaeological Laboratory, the Henan Provincial Institute of Cultural Relics and Archaeology, using the MS-2 magnetic susceptibility system (Bartington Instruments). Ten grams of each sample (loose and dry) are taken and tightly covered using plastic wrap. Each sample is tested three times at the high and low frequencies, respectively, and the averages of the three tests are taken as the final results to calculate the mass and frequency of magnetic susceptibility.

## Results

### Dating results and the chronological framework of the sedimentary section

The AMS ^14^C dating results are consistent with the archaeological dates and field stratigraphic analysis (**Table 1**). For the west wall section of T7, Layer ⑤ is attributed to the late Longshan culture; Layer ④ is dated back to the Xia-Shang Dynasty; Layer ③ represents the deposits of the Zhou Dynasty.

**Table 1:** Dating results.

Sample	Lab. N	Material	Measured Radiocarbon Age	Calibration	
				1σ (68.2%)	2σ (95.4%)
Layer③ of T7	Beta-602064	organic matter	2620 ± 30 BP	2758 (68.21%) 2737CalBP	2771 (95.41%) 2722CalBP
Layer ④ of T7	Beta-602065	organic matter	3380 ± 30 BP	3681 (9.9%) 3668CalBP 3639 (58.24%) 3572CalBP	3694 (90.72%) 3555CalBP 3528 (3.25%) 3506CalBP 3504 (1.42%) 3493CalBP
Layer ⑤ of T7	Beta-602066	organic matter	3980 ± 30 BP	4514 (35.98%) 4481CalBP 4443 (32.15%) 4415CalBP	4525 (90.72%) 4402CalBP 4367 (3.25%) 4357CalBP 4320 (0.5%) 4312CalBP

**Figure 3:**
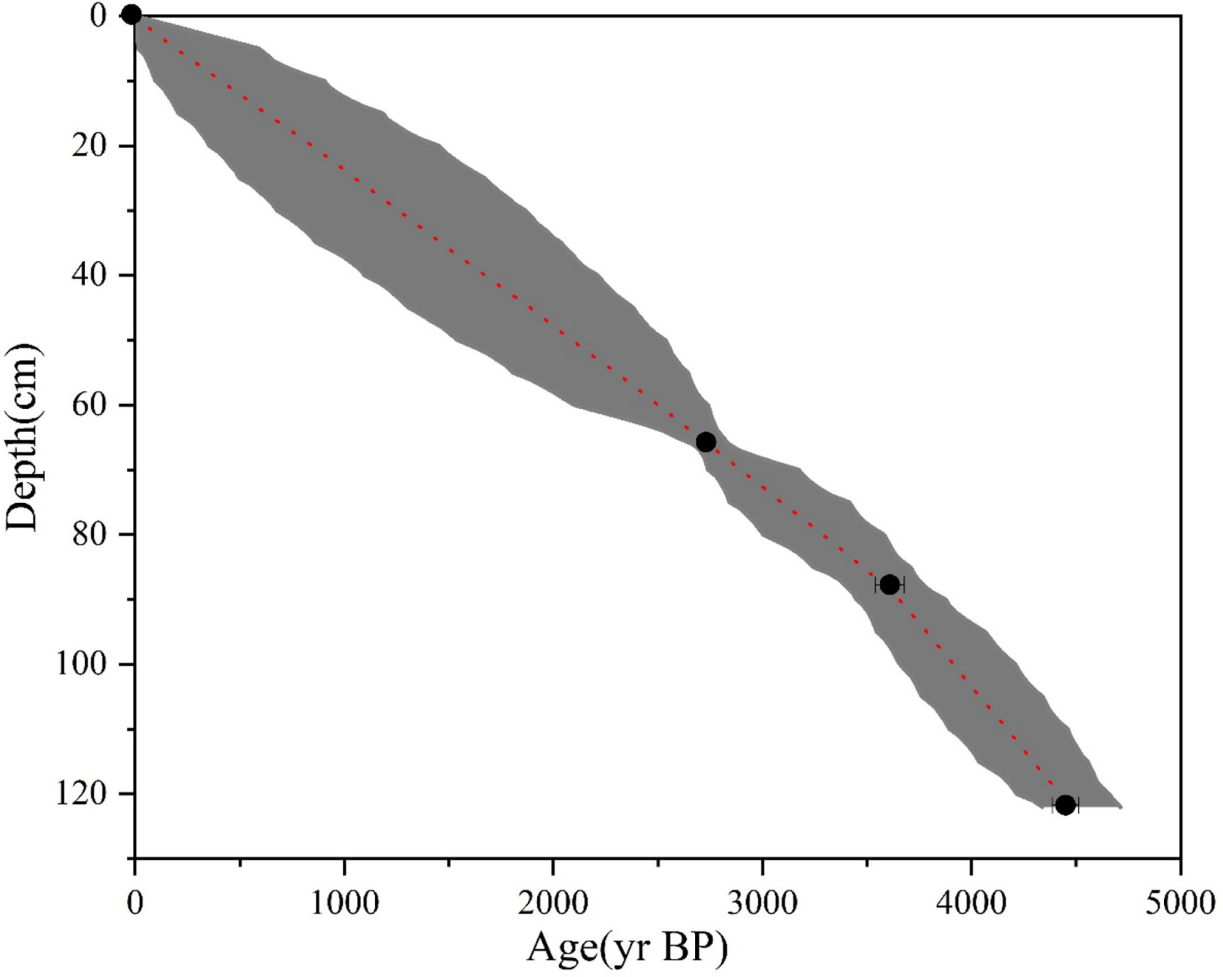
The depth-age model of the T7 west wall section.

The depth-age model of the T7 west wall section shows that Layers ①–⑤ are attributed to the modern age, Ming-Qing Dynasty, Eastern Zhou Dynasty, Shang Dynasty, and Longshan culture, respectively (**Figure 3**). Besides, for the T13 west wall section, Layers ①–③ that are more affected by human activities are attributed to the modern age, Ming-Qing Dynasty, and Shang Dynasty-Longshan culture, respectively. The attributes of Layer ⑥ of the T7 section are consistent with those of Layer ④ of the T13 section which is dominated by sands. Such deposits are commonly distributed in the southern Taihang piedmont and represent the flooding deposition during the formation of the alluvial plain. Layer ⑦ of the T7 section are typical results of fluvial processes. Considering the age-depth model, these deposits shall be formed at 6–11 ka BP. The medium-coarse sands below Layer ⑦ of the T7 section and Layer ④ of the T13 section are believed to be formed at the base of the alluvial fan during the late Pleistocene.

### Pollen assemblage

The pollen analysis indicates that the sedimentary samples from the FTG site all present very low pollen concentrations, with a maximum of about 68 spores/g and a minimum of only 5 spores/g. Low pollen concentrations are relatively common in the region. This phenomenon also appears in many geological sections in the immediate vicinity, such as the Shiyuan section and the Dahecun section (Xu et al., 2013; Ren et al., 2021).

**Figure 4:**
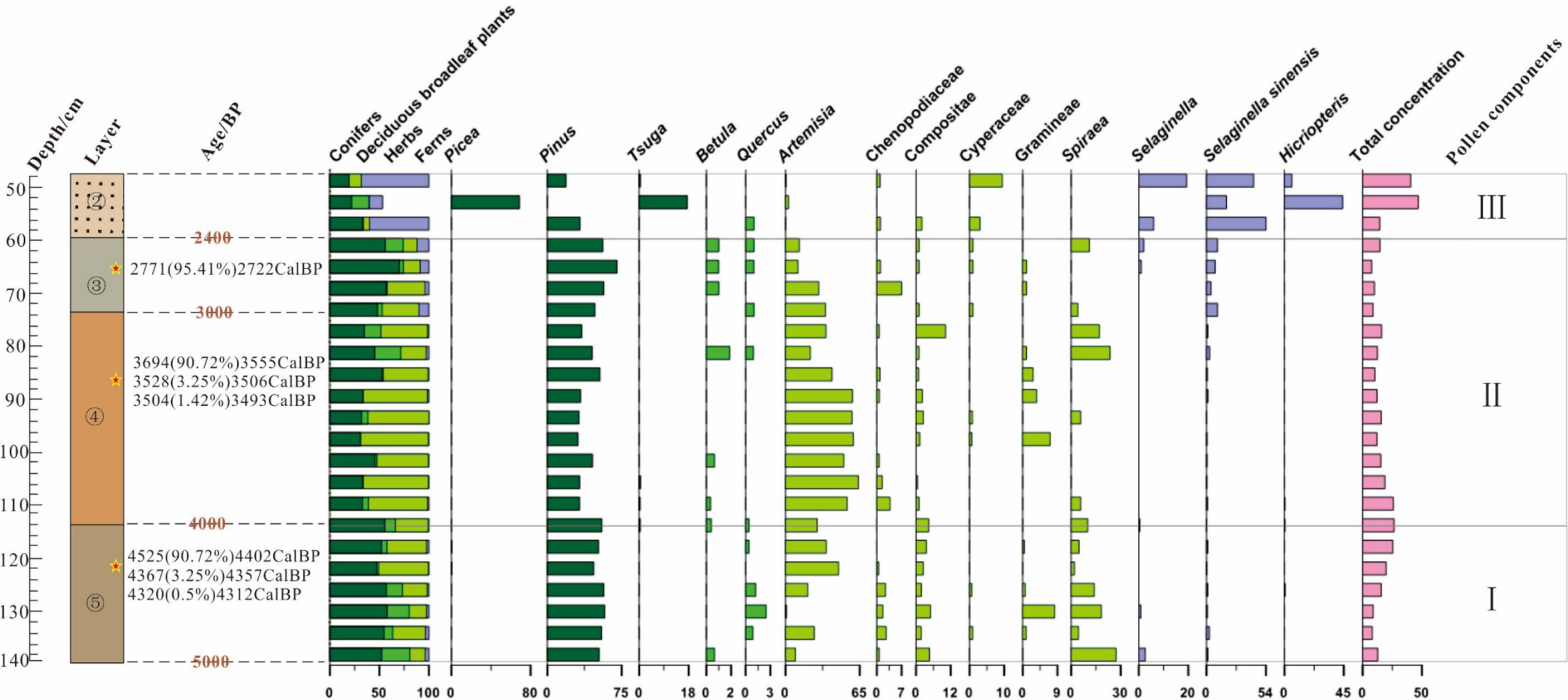
Palynogram of the FTG site.

Deposits below Layer ⑤ have an extremely low content of pollen. The pollen types identified across the geological section are also limited and a total of 45 families are observed (**Figure 4**). Specifically, the needle-leaf plant includes three families (genera), namely *Picea*, *Pinus*, and *Tsuga*; the deciduous broadleaf plant includes 15 genera, namely *Rhus*, *Betula*, *Carpinus, Ulmus, Lonicera, Viburnum, Salix, Tilia, Quercus, Ericaceae, Spiraea, Castanea, Juglans, Oleaceae*, and *Nitraria*; the herbaceous plant includes 19 families (genera), namely *Ephedra, Boraginaceae, Artemisia, Caryophyllaceae, Chenopodiaceae, Compositae, Gentiana, Convolvulaceae, Gramineae, Cultural Gramineae, Saxifragaceae, Potentilla, Urtica, Polygonum, Sanguisorba, Euphorbiaceae, Scrophulariaceae, Cyperaceae*, and aquatic *Typha*; the fern includes six families (genera), namely *Lycopodium, Selaginella. Sinensis, Hicriopteris, Polypodiaceae, and Angiopteris*. Moreover, a few freshwater still-water algae are observed, including *Concentricystes* and *Zygnema*.

According to the pollen concentration and its variation characteristics, three components are identified in the section.

**Assemblage III:** *Selaginella Sinensis*-*Pinus*-*Sellaginella*-*Cyperaceae* corresponds to the lower part of Layer ② of the T7 section (at depths of 48–60 cm).

The total pollen concentration of this assemblage is 14–16 grains/g. The fern pollen is the most abundant (60.0%–68.83%), followed by pollen grains of needle-leaf plants (17.53%–33.0%), which is further followed by the fewest pollen grains of herbaceous plants (6.0%–13.64%) and deciduous broadleaf plants (0–1.0%). The pollen grains of needle-leaf plants are only of *Pinus* (17.53%–33.0%), while those of the deciduous broadleaf plant are only of *Quercus*. In terms of the herbaceous plant, the pollen grains are seen with a predominance of those of *Cyperaceae*, followed by the pollen grains of *Compositae* (0–2.60%) and *Chenopodiaceae* (0–1.0%); only a few are attributed to the *Caryophyllaceae*, *Artemisia*, *Euphorbiaceae*, and *Polygonum*. The spores of the fern are mostly contributed by the *Selaginella Sinensis* (42.92%–54.0%). The second-largest contributor is *Sellaginella* (6.0%–19.34%), followed by the third *Hicriopteris* (0–6.49%). The spores of *Angiopteris* and other families (genera) are rare.

**Assemblage II**: *Pinus*-*Artemisia*-*Spiraea*-*Compositae*-*Selaginella Sinensis* corresponds to Layers ③ and ④ of the section (at depths of 60–112 cm).

The totalpollen concentration of this assemblage is 7–25 grains/g. The needle-leaf plant has the most pollen grains (30.97%–70.30%), followed by those of the herbaceous plant (14.0%–68.39%), the fewer pollen grains of the deciduous broadleaf plant (0–26.42%), and also the fewer spores of fern (0–12.0%). The pollen grains of needle-leaf plants are found with a predominance of those of *Pinus* (30.97%–70.30%), accompanied by a few pollen grains of *Tsuga*. For the deciduous broadleaf plant, the pollen grains of *Spiraea* are occasionally seen with high contents and a few pollen grains of *Betula*, *Carpinus*, *Nitraria*, *Lonicera*, and *Viburnum*. *Quercus*, *Tilia*, *Ulmus*, and *Oleaceae* are observed. Among herbaceous plants, *Artemisia* presents the most pollen grains (10.89%–63.78%), followed by *Compositae* (0–10.20%), *Chenopodiaceae* (0–7.0%), *Gramineae* (0–7.10%), and *Cyperaceae* (0–1.0%); the pollen grains of *Ephedra*, *Convolvulaceae*, *Gentiana*, *Sanguisorba*, *Saxifragaceae*, and aquatic *Typha* are seldom seen. In terms of the fern, most spores are attributed to *Selaginella Sinensis* (0–10.0%), with some from *Sellaginella* and *Hicriopteris*.

**Assemblage I:** *Pinus*-*Artemisia*-*Spiraea*-*Gramineae* corresponds to Layer ⑤ of the section (at depths of 112–138 cm).

This assemblage is found with a total pollen concentration of 8–26 grains/g, composed of the most pollen grains of needle-leaf plants (47.26%–57.85%), those of herbaceous plants (15.23%–50.25%), then those of deciduous broadleaf plants (1.99%–28.48%), and at last the fewest spores of fern (0.50%–3.97%). Specifically, *Pinus* is dominant among needle-leaf plants, with some pollen attributed to *Picea* and *Tsuga*. For the deciduous broadleaf plant, *Spiraea* presents the most pollen (1.99%–27.15%), while that of *Quercus* (0–2.48%), *Rhus*, *Betula*, *Carpinus*, *Ericaceae*, *Castanea*, *Juglans*, *Salix*, *Tilia*, and *Ulmus* is rarely seen. For the herbaceous plant, the pollen of *Artemisia* is the most (0.83%–46.27%), successively followed by the less pollen of *Gramineae* (0–8.26%), *Compositae* (1.74%–4.96%), *Chenopodiaceae* (0–2.61%), *Boraginaceae*, *Cyperaceae, Cultural Gramineae, Polygonum, Scrophulariaceae, Potentilla, Urtica, Saxifragaceae*, and aquatic *Typha*. For the fern, only a few spores of *Selaginella* (0–2.65%), *Selaginella Sinensis* (0–2.61%), *Lycopodium*, *Polypodiaceae*, *Hicriopteris*, and *Angiopteris* are identified.

### Grain size distribution

Grain size distribution is closely related to the dynamic conditions and sedimentary environment during deposition. It is dependent on the rainfall, transportation dynamics, and geomorphic position, with its variation implying the relative strength of dynamic conditions and surface process evolution (Xia et al., 2003; Wang et al., 2015, Wang et al., 2016, 2017; Rosen, 2008).

**Figure 5:**
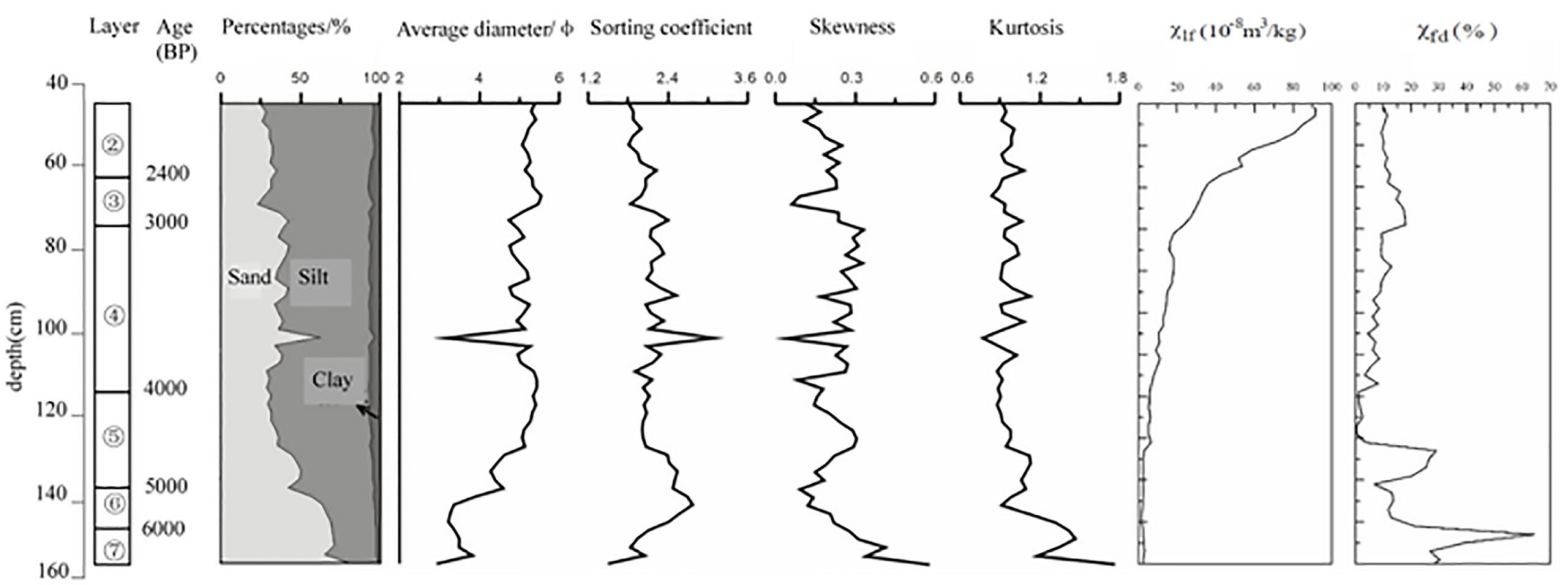
Grain size and magnetic susceptibility of the FTG site.

Layer ⑦ of the T7 west wall section is dominated by sands, followed by silts. Layer ⑥ is composed of medium-grained sands and represents an obvious erosional event. Grain size analysis is not implemented in Layer ⑥ since the grains of this layer are generally large. For Layer ⑤ and above, the sediment grains are seen with a predominance of silts, followed by sands; furthermore, the younger sediments have higher clay contents (**Figure 5**).

Layer ⑥ is primarily dominated by sands (80.4%–56.6%), the content of which gradually declines vertically upward. The second-largest component is silt (18.3%–39.6%) and the clay content is very low (1.2%–3.9%). The average grain size is 2.9–3.9Φ. The sorting coefficient is 1.5–2.7 and the kurtosis is 0.9–1.7, indicating poor sorting. The skewness is 0.58–0.14, suggesting the presence of coarse-grained components.

Layer ⑤ consists of silts (45%–64%), upward-decreasing sand (28%–50%), and upward-increasing clay (3.5%–6.9%). The average grain size is 4.3–5.4Φ. The sorting coefficient is 2.5–2.0, which, together with the medium kurtosis, implies weakening hydrodynamics and steadily developing limnetic facies. The sorting is relatively good. The skewness is 0.1–0.3 (positively skewed) and suggests the presence of certain coarse components.

Layer ④ is dominated by silts (51%–65%), followed by sands (28%–42%) and clays (5.7%–6.5%). There is a sharp increase in the sand content in the middle of the layer, which may result from a flooding event. The average grain size is 4.8–5.4Φ; the sorting coefficient is 1.9–2.5; the kurtosis is medium (0.9–1.1); the skewness is 0.1–0.3 (positively skewed). In general, the hydrodynamics of this period are weak and the sediments are well sorted. This layer shall represent the most stable development of the limnetic facies.

Layer ③ is dominated by silts (52%–72%), followed by sands (23%–42%) and clays (4.2%–7.1%). The average grain size is 4.7–5.5Φ. The sorting coefficient is 1.8–2.4 and the kurtosis is 0.9–1.1 (medium), which indicates stable hydrodynamics and good sorting. The skewness is 0.1–0.2 (positively skewed). The development of the limnetic facies is relatively stable during this period.

Layer ② is still dominated by silts (63%–71%), followed by sands (25%–32%) and clays (3.7%–5.6%). The site area had completely changed into a terrestrial environment by the deposition of Layer ②.

### Magnetic susceptibility

Can be used as a proxy during the paleo-environmental reconstruction (Ji and Xia, 2007). It is relatively low in the fluvial-lacustrine facies (below Layer ⑤), and it gradually increases vertically upward, reaching high values in Layers ③ and ② (**Figure 4**). This is attributed to the intensifying human activity. The lower half of Layer ⑤ has high and significantly variable magnetic susceptibility, which results from the abundant superparamagnetic grains in the sediment.

For Layer ⑤, the mass magnetic susceptibility is (2.5–6.6) ×10^-8^ m^3^/kg, while the frequency magnetic susceptibility varies greatly within the range of (1.1–29.1) ×10^-8^ m^3^/kg.

For Layer ④, the mass magnetic susceptibility grows gradually within the range of (6.9–18.2) ×10^-8^ m^3^/kg, while the frequency magnetic susceptibility changes within the range of (3.4–13.0) ×10^-8^ m^3^/kg.

For Layer ③, the mass magnetic susceptibility widely ranges from 23.4 ×10^-8^ m^3^/kg to 36.1 ×10^-8^ m^3^/kg, whereas the frequency magnetic susceptibility is relatively stable, with a range of (18.7–17.1) ×10^-8^ m^3^/kg.

For Layer ②, the mass magnetic susceptibility varies in the range of (42.7–91.5) ×10^-8^ m^3^/kg, whereas the frequency magnetic susceptibility is (9.4–12.5) ×10^-8^ m^3^/kg.

## Discussion

### Reconstruction of the Holocene vegetation landscape

There is a set of pollen data from the Wangjiadian (WJD) section (36°5′47.66″N, 114°24′5.52″E) in the adjacent Anyang area. It is a 198 cm long continuous and undisturbed section from a location with no cultural remains (Cao et al., 2010). According to the stratigraphic chronology, our Assemblages I, II, and III should correspond to the Pollen Zones 4, 5, and 6 of the WJD section, respectively. The pollen in FTG and WJD sections is found to have very similar species and quantities. Although the FTG section is imprinted by the human culture sediment, the pollen results are still representative of the region.

The early-middle Holocene sediment in the study area contains little pollen. Although this phenomenon is closely related to the type of sediments, it also to some extent indicates the scarce vegetation in the floodplain during the early-middle Holocene.

During 5000–4000 BP, mixed broadleaf-conifer forest-grassland is dominant. The needle-leaf tree is mainly *Pinus*, while the deciduous broadleaf plant is dominated by *Spiraea*. Moreover, the herbaceous plant is seen with a predominance of *Artemisia*, *Gramineae*, and *Compositae*. Previous studies on pollen grains of *Pinus* indicate that pine forests are likely, only if the pollen content of *Pinus* is over 30% (Li and Yao, 1990; Li et al., 2005; Xu et al., 2007). This region is associated with a *Pinus* pollen content of 46.77%–57.85%, suggesting the possible presence of pine forests in the surrounding areas. Water is suggested to be nearby according to the presence of heliophilous trees such as *Rhus*, *Carpinus*, *Juglans*, and *Tilia*, the considerably higher pollen content of *Artemisia* than that of *Chenopodiaceae*, and the few pollen grains of aquatic *Typha*. Identified pollen of *Cultural Gramineae* is likely to be indicative of human activities and crop cultivation. The terrestrial plant assemblage of *Pinus*-*Artemisia*-*Spiraea*-*Gramineae* reveals that the region has changed from floodplain to land and large-scale human activities have started in this region.

During 4000–2400 BP, the mixed broadleaf-conifer forest-grassland is prevalent. The overall content of the deciduous broadleaf plant is lower than that of Assemblage I. The terrestrial vegetation assemblage of *Pinus*-*Artemisia*-*Spiraea*-*Compositae*-*Selaginella Sinensis* implies the growing area of land. Meanwhile, sporadic aquatic plants suggest the presence of some large waters in the study area.

During the Ming-Qing Dynasty, the open forest-grassland takes dominance. Ferns extensively grow under trees. The abundance of woody plant pollen is notably lower than that of the former two periods; in contrast, the spores of fern (*Selaginella* and *Selaginella Sinensis*) are much higher during this period. The content of *Pinus* pollen is less than 30% at the interval of 48–56 cm, which means the absence of pine forests during this period. The representative vegetation assemblage is *Selaginella Sinensis*-*Pinus*-*Sellaginella*-*Cyperaceae*, accompanied by no aquatic plants.

### Holocene environmental evolution in the study region

**Figure 6:**
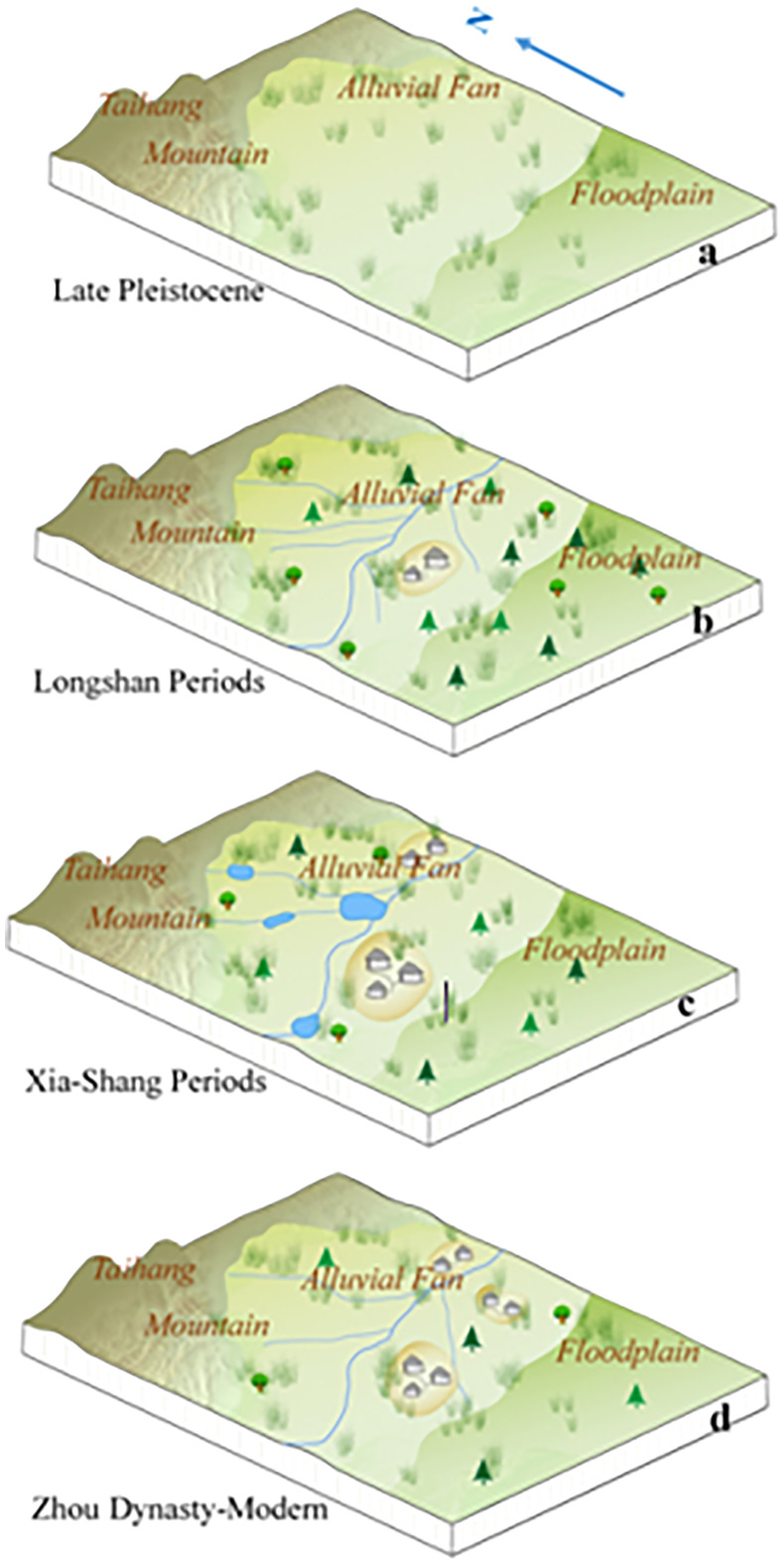
Regional landscape evolution **(A)**. In the Late Pleistocene (before 10000 BP), there were no fixed channels on the alluvial fan, with dominant overland flow. The vegetation landscape was mainly grassland; **(B)** During the Longshan period (4000-4500 BP), rivers erode down to form fixed channels on the alluvial fan. The pollen assemblage of *Pinus*-*Artemisia*-*Spiraea*-*Gramineae* reflects the vegetation landscape of the forest-steppe. Humans began to settle down there; **(C)** During the Xia-Shang periods (4000-3100 BP), many lakes formed on the alluvial fan. The vegetation landscape is still forest-steppe. Human activity gradually intensifies; **(D)** During the Zhou Dynasty (3100 BP-), the lakes disappeared. The vegetation landscape was thin grassland again.).

Holocene environmental evolution in the study region can be clarified (**Figure 6**), according to the vegetation landscape evolution, sedimentary characteristics, grain size distribution, and magnetic susceptibility of the field geological sections.

From the end of the late Pleistocene to the early-middle Holocene, the study region is found with deposition of mostly grey-yellow silty-fine sands, which are basal sediments of the floodplain and have a very wide distribution. The sand content gradually declines vertically upward, accompanied by a certain amount of silts and a limited content of clays. The poor sorting is believed to result from flooding. As the region is located in the piedmont of Taihang Mountain, river systems are highly developed in this region, associated with strong hydrodynamics and short flow distance. Moreover, the water flow path is not fixed, featured by bifurcation and avulsion. Consequently, the floodplain presents a rather unstable landform.

During 6000–5000 BP, Layer ⑥ of the T7 section develops dark-brown medium-coarse grained sands with a trough-like distribution. These medium-coarse sands indicate high transportation capability of rivers and shall be lag deposits of river erosion. In contrast, the overlying loess-like silty-fine sands of the floodplain facies indicate the decreasing transportation capability of rivers. The sedimentary characteristics suggest that there once exists a fluvial erosion and deposition process—first, a wide gully is formed as the river erodes the early floodplain and then silty-fine sands accumulate in the gully.

During the late Longshan culture 5000–4000 BP, the grey-brown clay of Layer ⑤ of the T7 west wall section is deposited (representing the limnetic deposition). A large-scale river erosion event happened in the early stage of this period. The previous loess-like deposits are eroded into elongated downlands. After the river erosion, the silting-up of the river starts, and the limnetic facies begins to develop.

During 4000–3000 BP, the limnetic facies in the region continues developing. The color, lamination, and grain size distribution of the sediments are all similar to those of the lower sediments, except that the sediment color becomes lighter. No notable discontinuity of deposition is observed. The dominant presence of silts and the stable content of clays suggest weak hydrodynamics and steadily developing limnetic facies.

After 3000 BP, the limnetic facies remains in this region, but it tends to decrease and even vanish as suggested by the stable hydrological conditions according to the dominant presence of silts and the stable content of clays. With silting-up, the water depth decreases and the water area expands to the edge of the loess downland. Afterward, the limnetic facies is gone and pedogenesis begins. The magnetic susceptibility is considerably higher during this period, indicating the intensifying human activities in this region.

### Human activity history and evolution in the region

Although our archaeological excavation area is a bit small, the artificial remains in different layers can still reveal some characteristics of regional human activities (**Table 2**). Through these remains, we confirm that large-scale human activities in the piedmont of Taihang Mountain start in the late Longshan culture (about 4000 BP). After an erosional event, stable river channels are formed and the floodplain gradually changes into the mixed broadleaf-conifer forest and grassland. Humans start to dwell in the highlands, and they get stable water supply from nearby gullies formed by river erosion.

**Table 2:** The main artificial remains found in archaeological excavation.

Culture	Artificial Remains
Longshan	About 10 ash pits.
Xia-Shang	About 10 ash pits and a tomb.
Zhou	About 10 ash pits and 5 tombs

During the Xia-Shang Dynasty (4000–3100 BP), gullies continue growing and the overall landform is stable. Ancient humans still live in the highlands besides the gullies where the water supply can be guaranteed.

During the Zhou-Han Dynasty (3100–2000 BP), the downward erosion of rivers in the region results in the vanishment of the limnetic facies in the gullies. Consequently, humans have to expand their activity area.

Since 2000 BP, the limnetic facies vanishes, accompanied by the prevalence of open forests and grasslands. Human activities further grow during this period.

The fixation of river channels and the transition of the region from the floodplain to the open forests and grasslands are exemplified to be vital for the large-scale human cultural development in the piedmont. The massive shrinkage of the floodplain since the late Longshan culture contributes to the favorable living environment for human settlements. Afterward, the region is associated with large-scale human activities and the human culture continuously develops.

Archaeological excavations at some nearby sites have also revealed a similar history of regional human activities. In the Huixian Mengzhuang site, the only found early remains are ash pits (Henan Provincial Institute of Cultural Relics and Archaeology, 1999). During the Longshan period, there were a large number of houses in this site. Archaeologists have even found a city site (Henan Provincial Institute of Cultural Relics and Archaeology, 2000). In the Xinxiang Lidazhao site, all remains of the Yangshao culture are ash pits. Since the Longshan period, the remains have become more and more abundant in this site (Han and Zhao, 2006). In the Huixian Suncun site, houses, wells, and ash pits of the Shang Dynasty are found (Archaeology Department of History School of Zhengzhou University et al., 2008). These indicate that human activities have gradually intensified since the Longshan period.

Our research results are consistent with the regional human activity history from the summed probability distribution (SPD) of large radiocarbon databases (Wang et al., 2014; Ren et al., 2021). According to an SPD of radiocarbon dates in Central China (Ren et al., 2021), the regional population in the late Longshan period (4000 BP) is higher than that in previous periods. Then the regional prehistoric culture continues to develop and reaches its peak in the Xia and Shang Dynasties. These also further prove the rationality of our research results.

## Conclusions

The history and environmental mechanism of human activities in the piedmont zone of Taihang Mountain, a well-known mountain with high ecological and cultural significance in history, are of great importance to reveal the interaction between humans and environments. The following conclusions are drawn in this research:

First, the southern Taihang piedmont is dominated by floodplains during the Late Pleistocene. During the late Longshan culture (4500–4000 BP), the region is found with the pollen assemblage of *Pinus-Artemisia-Spiraea-Gramineae*, with alternating open forest-grassland and rivers/lakes. This state remains until 2000 BP. Afterward, lakes and marshes vanish, and the region is dominated by the open forest-grassland.

Second, a regional fluvial erosional event occurs during the middle Holocene (6000–4500 BP), which leads to the gradual fixation of river channels to form stable flow paths. Therefore, the situation with the unfavorable long-term flooding in the region is finally changed.

Third, the large-scale human activities in the southern Taihang piedmont start at about 4000 BP during the late Longshan culture. The fixation of river channels and the prevalence of open forest-grassland have played important roles in this process.

This research validates the fundamental role of the natural environment in impacting the interaction between humans and the local environment. Only if the natural environment becomes favorable can large-scale human activities be sustained to subsequently create the brilliant early civilization.

## Data availability statement

The original contributions presented in the study are included in the article/supplementary material. Further inquiries can be directed to the corresponding authors.

## Author contributions

XZ designed the research. XZ and PL performed the research and completed writing. XZ, PL, and YL analyzed the data. PC and YL drawed pictures. SY provided archaeological context on the site. ZX directed field surveys. All authors contributed to the article and approved the submitted version.

## Funding

The study is funded by the National Key R&D Program of China (2020YFC1521900 and 2020YFC1521904), the National Natural Science Foundation of China (41971016, 41671014 and 42071119), the Basic Scientific Fund for Henan Provincial Research Institutes (2014HAJB-KG05) and the Digital Environment Archaeology Specially-appointed Researcher of Henan, China (210501002).

## Conflict of interest

The authors declare that the research was conducted in the absence of any commercial or financial relationships that could be construed as a potential conflict of interest.

## Publisher’s note

All claims expressed in this article are solely those of the authors and do not necessarily represent those of their affiliated organizations, or those of the publisher, the editors and the reviewers. Any product that may be evaluated in this article, or claim that may be made by its manufacturer, is not guaranteed or endorsed by the publisher.
